# Predictive Factors for HBsAg Seroconversion Following Acute Hepatitis B Virus Infection: A Multicenter BUHASDER Study

**DOI:** 10.3390/v17091182

**Published:** 2025-08-29

**Authors:** Ozge Caydasi, Derya Öztürk Engin, Ömer Karaşahin, Ayşe Batırel, Süleyman Dolu, Müge Toygar Deniz, Sıla Akhan, Kemalettin Özden, Selma Tosun, Burak Sarıkaya, Levent Görenek, Merve Çelik Aydoğdu, Eyüp Arslan, Fatma Yılmaz Karadağ, Serpil Erol, Semiha Çelik Ekinci, Ahmet Şahin, Müge Özgüler, Pınar Yürük Atasoy, Derya Seyman, Süleyman Yıldırım, Gözde Derviş Hakim, Tuba Damar Çakırca, Özlem Bayraktar Saral, Tuğba Sarı, Türkkan Öztürk Kaygusuz, Süleyman Günay, Şükran Köse

**Affiliations:** 1 Department of Infectious Diseases and Clinical Microbiology, Sancaktepe Education and Research Hospital, Health Sciences University, 34785 Istanbul, Turkey; dr.deryaengin@gmail.com (D.Ö.E.); merve.tsche@gmail.com (M.Ç.A.); dreyuparslan@hotmail.com (E.A.); dr_fatma@hotmail.com (F.Y.K.); 2 Department of Infectious Diseases and Clinical Microbiology, Erzurum Education and Research Hospital, Health Sciences University, 25070 Erzurum, Turkey; mrkrshn@hotmail.com; 3 Department of Infectious Diseases and Clinical Microbiology, Kartal Dr. Lütfi Kırdar City Hospital, Health Sciences University, 34865 Istanbul, Turkey; aysebatirel@yahoo.com; 4 Department of Gastroenterology, Faculty of Medicine, Dokuz Eylül University, 35340 Izmir, Turkey; suleyman.dolu@deu.edu.tr; 5 Department of Infectious Diseases and Clinical Microbiology, Faculty of Medicine, Kocaeli University, 41380 Kocaeli, Turkey; mugedeniz90@gmail.com (M.T.D.); silaakhan@gmail.com (S.A.); 6 Department of Infectious Diseases and Clinical Microbiology, Faculty of Medicine, Atatürk University, 25240 Erzurum, Turkey; ozdenkemal@gmail.com; 7 Department of Infectious Diseases and Clinical Microbiology, Izmir City Hospital, Health Sciences University, 35170 Izmir, Turkey; selma.tosun@yahoo.com; 8 Department of Infectious Diseases and Clinical Microbiology, Sultan 2. Abdulhamid Han Education and Research Hospital, Health Sciences University, 34668 Istanbul, Turkey; burak_tibbiyeli@hotmail.com (B.S.); gorenekl@gmail.com (L.G.); 9 Department of Infectious Diseases and Clinical Microbiology, Haydarpaşa Numune Education and Research Hospital, Health Sciences University, 34668 Istanbul, Turkey; sererol@gmail.com; 10 Department of Infectious Diseases and Clinical Microbiology, Fatih Sultan Mehmet Education and Research Hospital, Health Sciences University, 34752 Istanbul, Turkey; dr_semiha@hotmail.com; 11 Department of Infectious Diseases and Clinical Microbiology, Faculty of Medicine, Dr Ersin Arslan Education and Research Hospital, Gaziantep Islam Science and Technology University, 27010 Gaziantep, Turkey; ahmet27sahin@hotmail.com; 12 Department of Infectious Diseases and Clinical Microbiology, Elazığ Fethi Sekin City Hospital, Health Sciences University, 23280 Elazig, Turkey; mugeozguler@gmail.com; 13 Department of Infectious Diseases and Clinical Microbiology, Ankara Bilkent City Hospital, Health Sciences University, 06800 Ankara, Turkey; pinaryuruk@windowslive.com; 14 Department of Infectious Diseases and Clinical Microbiology, Antalya Education and Research Hospital, Health Sciences University, 07100 Antalya, Turkey; seymander@gmail.com; 15 Department of Gastroenterology, Izmir City Hospital, Health Sciences University, 35170 Izmir, Turkey; deu.syldrm@gmail.com (S.Y.); gozdedervis@gmail.com (G.D.H.); 16 Department of Infectious Diseases and Clinical Microbiology, Health Sciences University, Şanlıurfa Education and Research Hospital, 63300 Sanliurfa, Turkey; dr.tubadamar@gmail.com; 17 Department of Infectious Diseases and Clinical Microbiology, Trabzon Kanuni Education and Research Hospital, Health Sciences University, 61040 Trabzon, Turkey; lembayraktar@gmail.com; 18 Department of Infectious Diseases and Clinical Microbiology, Faculty of Medicine, Pamukkale University, 20070 Denizli, Turkey; tsari@pau.edu.tr; 19 Department of Infectious Diseases and Clinical Microbiology, Faculty of Medicine, Fırat University, 23119 Elazig, Turkey; turkkan69@gmail.com; 20 Department of Gastroenterology, Faculty of Medicine, İzmir Katip Çelebi University, 35620 Izmir, Turkey; suleyman.gunay@gmail.com; 21 Department of Infectious Diseases and Clinical Microbiology, Faculty of Medicine, Dokuz Eylül University, 35340 Izmir, Turkey; sukrankose@yahoo.com

**Keywords:** acute hepatitis B, entecavir, HBsAg seroconversion, predictor, tenofovir

## Abstract

Experience with the effect of antiviral therapy on HBsAg seroconversion in acute hepatitis B is limited. We aimed to evaluate the factors affecting HBsAg seroconversion in patients with acute hepatitis B (AHB) receiving antiviral treatment. We performed a retrospective and multicenter study involving 107 adult patients who received antiviral treatment for AHB between January 2018 and December 2023. The median age was 48 (min–max, 18–91) years, and 66.3% of the patients were male; 70 patients with 24-week follow-up (15 patients without HBsAg seroconversion versus 55 patients with HBsAg seroconversion) were compared based on HBsAg seroconversion status. Multivariate logistic regression analysis revealed that age was independently associated with HBsAg seroconversion (odds ratio = 0.926; 95% confidence interval (CI) 0.874 to 0.981; *p* = 0.009). A one-year increase in age was associated with a 0.926-fold decrease in HBsAg seroconversion. In conclusion, age was an independent predictor of HBsAg seroconversion following acute hepatitis B virus infection.

## 1. Introduction

Acute hepatitis B virus (HBV) infection presents a matter of concern, as approximately 5% of patients develop chronic infection and 0.1–1% progress to acute liver failure (ALF), significantly reducing survival [1,2]. HBV can be transmitted by unprotected sexual contact, illicit drug use, different percutaneous needle interventions such as acupuncture, piercings, or tattoos, and from mother to child, particularly in developing countries [1]. Acute hepatitis B (AHB) is estimated to be approximately 1.2 million in 2022, although the incidence has decreased over the years [3].

AHB may present with a wide range of clinical manifestations, from subclinical or anicteric forms to fulminant hepatitis [4]. ALF is characterized by a rapid decline in liver function, altered mental status, and coagulopathy in the absence of prior liver disease [5]. HBV-related ALF remains unclear, but it may be associated with an enhanced immune response to HBV replication [6]. HBV-related ALF patients without orthotopic liver transplantation have a poor prognosis, with survival rates ranging from 19% to 33% [7].

Acute hepatitis B mainly improves with supportive care, but in some individuals, antiviral therapy has been recommended to prevent progression to a fulminant course [8,9]. Additionally, alternative treatments such as baicalein and baicalin show promising potential for HBV, with lower toxicity and improved therapeutic efficacy [10].

The experience with antiviral treatment in AHB remains insufficient. There are observational studies and case reports in the literature, mainly with lamivudine, involving a limited number of patients [6,11]. There are different results regarding the effect of antiviral therapy on the need for liver transplantation and mortality in AHB [6,12]. There is also no consensus regarding the impact of AHB treatment on chronicity [13,14]. In this study, we aimed to determine the predictive factors for hepatitis B surface antigen (HBsAg) seroconversion in patients receiving antiviral agents for acute hepatitis B.

## 2. Material and Methods

This is a retrospective, multicenter study of 107 patients aged ≥18 years who received antiviral treatment for AHB at 14 hospitals between January 2018 and December 2023. The diagnosis of AHB was based on recent onset of typical symptoms with elevated serum alanine aminotransferase (ALT) levels and positivity for serum HBsAg and IgM antibody to the hepatitis B core antigen (anti-HBc IgM) in the absence of a prior history of HBV infection [15]. Serum quantitative HBV-DNA testing by polymerase chain reaction (PCR) assay was performed for confirmation when available. AHB treatment was initiated according to current HBV guideline recommendations [9]. The patients who did not receive antiviral therapy during clinical follow-up were excluded from the study.

Clinical and laboratory data were collected using hospital electronic records, including age, gender, underlying diseases such as diabetes mellitus (DM), hypertension (HT), coronary artery disease (CAD), neurologic disease, chronic obstructive pulmonary disease (COPD), and malignancy; smoking; alcohol consumption; drug abuse; prior hepatitis B vaccination status; symptoms at hospital admission; serological test results; and ultrasound findings. Serological tests were performed using commercially accessible enzyme-linked immunoassays. Laboratory values, including hemogram, liver function test, and coagulation parameter, were recorded at the initiation of antiviral therapy. Antiviral agents, indications for treatment, total duration of antiviral therapy, and supportive care were recorded.

ALF was defined as the development of encephalopathy and coagulopathy (international normalized ratio (INR) ≥ 1.5) in a patient with severe liver injury, in the absence of pre-existing liver disease and with an illness duration of less than 26 weeks [5]. Hepatic encephalopathy (HE) was classified into grades 1–4 according to the West Haven criteria [16]. The liver transplantation status and outcomes of each patient were recorded.

The patients were compared based on HBsAg seroconversion status. HBsAg seroconversion was defined as the loss of HBsAg and the presence of protective titers of hepatitis B surface antibody (anti-HBs) levels. This comparison included 70 patients with at least 24 weeks of follow-up. The patients with hepatitis A virus (HAV), hepatitis C virus (HCV), or human immunodeficiency virus (HIV) co-infections and other causes of liver injury and those who received immunosuppressive agents were excluded from this comparison.

### 2.1. Statistical Analysis

Data analysis was performed using the Statistical Package for the Social Sciences (SPSS) version 22.0 (SPSS Inc., Chicago, IL, USA). Descriptive data were presented as frequency distributions and percentages for categorical variables and as mean (±standard deviation) and median (with maximum and minimum values) for continuous variables. The chi-square test was used to compare categorical data between the groups, while the Kruskal–Wallis and Mann–Whitney U tests were performed to compare continuous data. A multivariate logistic regression analysis was conducted using statistically significant categorical and continuous variables affecting HBsAg seroconversion. (model: enter method; entry = 0.05, removal = 0.10). A *p*-value of less than 0.05 was considered statistically significant.

### 2.2. Ethical Approval

The study was approved by the Institutional Ethics Committee of Sancaktepe Training and Research Hospital on 12 June 2024 (Reference No. 176). The study was conducted in accordance with the principles outlined in the Helsinki Declaration.

## 3. Results

Acute hepatitis B virus infection was diagnosed in 107 patients with a median age of 48 (min–max, 18–91) years, and 71 (66.3%) of these patients were male. The most frequently observed comorbidity was HT (*n* = 22, 20.5%), followed by DM (*n* = 15, 14%), CAD (*n* = 14, 13%), and malignancy (*n* = 9, 8.41%). Thirty-six (33.6%) patients were active smokers, and 10 (9.34%) were social alcohol drinkers. There was no history of drug abuse. Prior hepatitis B vaccination status was unknown in 88 (82.2%) patients. Thirteen (12.1%) patients had not received any doses of the hepatitis B vaccine. Six (5.6%) patients did not respond to the hepatitis B vaccine. The most common complaints of the patients on admission were fatigue (*n* = 92, 85.9%), followed by loss of appetite (*n* = 73, 68.2%), nausea-vomiting (*n* = 61, 57%), and jaundice (*n* = 20, 18.6%). The median duration of the symptoms prior to antiviral treatment was 13 days (interquartile range (IQR), 8–20) (Table 1).

A total of 70 patients with AHB and 6-month follow-up were evaluated, of whom 55 (78.6%) patients had HBsAg seroconversion.

HBsAg seroconversion was statistically significantly lower in patients with advanced age and DM (*p* < 0.001, *p* = 0.034, respectively). HBsAg seroconversion was statistically significantly higher in the patients presenting with loss of appetite (*p* = 0.026) (Table 2).

Five patients (4.67%) had HIV co-infection, four (3.73%) had HAV co-infection, and three (2.8%) had HCV co-infection. Total hepatitis E virus (HEV) antibodies were evaluated in 35 patients and hepatitis delta virus (HDV) antibodies in 91 patients, and all results were found to be negative. Hepatitis B e antigen (HBeAg) was positive in 78.5% (84) of the patients. Abdominal ultrasound revealed hepatomegaly in 42 (39.2%) and hepatosteatosis in four (3.73%) patients. Prothrombin time (PT), INR, and aspartate aminotransferase (AST) levels were statistically significantly higher in the patients who achieved HBsAg seroconversion (*p* = 0.042, *p* = 0.011, and *p* = 0.048, respectively) (Table 3).

In total, 42 (39.2%) patients were initiated on antiviral treatment due to prolonged INR, 20 (18.6%) due to persistent symptoms and 15 (14%) due to hyperbilirubinemia. Antiviral treatment was initiated in 16 (14.9%) patients due to ALF. Forty-nine (45.7%) patients received tenofovir disoproxil fumarate (TDF), 41 (38.3%) patients received entecavir, 13 (12.1%) patients received lamivudine, and four (3.73%) patients received tenofovir alafenamide fumarate (TAF). The median duration of antiviral therapy was 32 (IQR, 28–90) days. Vitamin K was administered to 15 (14%) patients, and fresh frozen plasma was indicated in four (3.73%) patients (Table 1). HE was present in 16 patients (14.9%) at the initiation of antiviral therapy and progressed in five patients (4.67%). Twelve (11.2%) patients had grade 1 and four (3.73%) patients had grade 2 HE. Twenty (18.6%) patients were referred to a liver transplant center. Living-donor liver transplantation was performed in one (0.93%) patient. A patient with progressive HE who was awaiting a suitable donor died. An elderly patient died due to acute pulmonary edema. There were no statistically significant results in the comparison of antiviral treatment indications, antiviral agents, supportive care, and outcomes according to HBsAg seroconversion status (Table 4).

A multivariate logistic regression model was performed using age, loss of appetite, presence of diabetes mellitus, PT, and AST levels. Only prothrombin time was retained in the model, given that INR and PT reflect the same coagulation parameter and their simultaneous inclusion would introduce multicollinearity. Age was identified as an independent predictor for HBsAg seroconversion (odds ratio = 0.926; 95% confidence interval (CI) 0.874 to 0.981; *p* = 0.009). An increase of one year in age was associated with a 0.926-fold reduction in HBsAg seroconversion (Table 5).

## 4. Discussion

Acute hepatitis B remains a significant issue worldwide. According to the European Surveillance System 2022 data, AHB predominantly occurred in individuals aged 25–54 years and was almost twice as common in men [17]. The demographic data of the patients in this study were similar to the literature. Acute co-infections with multiple hepatitis viruses are relatively rare [18]. There have been case reports of acute co-infection with HBV in the literature [18,19]. In this study, 3.73% of AHB patients had HAV and 2.8% had HCV co-infections. HBV-HIV co-infections are also rare, with approximately 1% of HBV-infected patients being reported as co-infected with HIV [3]. In this study, the acute HBV-HIV co-infection rate was higher (4.67%). The primary source of transmission may be sexual contact in these patients. Intravenous drug use can also be an essential transmission route for co-infections [18]. However, the patients in our study had no history of drug abuse.

Antiviral treatment for acute hepatitis B has been practiced for over 25 years [20]. However, prospective randomized placebo-controlled trials for the efficacy of antiviral therapy in fulminant hepatitis B are ethically challenging to the current knowledge; there are primarily retrospective studies and case reports in the literature [21]. The European Association for the Study of the Liver (EASL) 2017 guideline recommends antiviral therapy for severe AHB patients with coagulopathy or a protracted course, such as persistent symptoms or jaundice for four weeks [9]. The American Association for the Study of Liver Diseases (AASLD) 2018 and Asia-Pacific hepatitis B guidelines have similar recommendations for the indications of antiviral therapy in AHB [8,22]. In this study, the recommendations of these guidelines were used to initiate antiviral treatment [8,9,22]. The most common indication for initiating antiviral therapy in these patients was a prolonged INR (39.2%). There were 16 patients in this study who received antiviral agents due to the development of ALF.

The effect of acute hepatitis B treatment on HBsAg seroconversion is a matter of concern. The faster decrease in viral load with antiviral treatment in AHB may result in insufficient presentation of HBV proteins to the immune system and potentially hinder antibody responses [23]. The literature presents different results regarding the HBsAg seroconversion rate in patients with AHB receiving antiviral treatment [13,14]. In previous studies conducted with lamivudine, the HBsAg seroconversion rate has been reported to vary from 10.14% to 76.9% in the patients [24,25,26]. These studies include a limited number of patients and show variability in follow-up duration. In recent years, experiences with high-potency nucleoside analogues in AHB treatment have been presented. Jochum et al. evaluated 32 patients with fulminant acute hepatitis B using antiviral treatment (21 entecavir, eight tenofovir, and three lamivudine) in the study, and HBsAg seroconversion was achieved in 72.7% of the patients [14]. In a study by Keser et al. in Turkey, the HBsAg seroconversion rate was found to be lower at 56.5% in 47 AHB patients who received TDF [13]. In a study conducted with 24 severe AHB patients who were candidates for liver transplantation, Özden et al. found that the HBsAg seroconversion rate was 73% in the patients receiving antiviral treatment (16 entecavir and eight TDF) [27]. In another study in Turkey, Gökçe et al. showed that HBsAg seroconversion was achieved in 100% of the patients in the severe AHB group using antiviral treatment (24 entecavir and one TDF) after 12 months of follow-up [21]. In our study, the HBsAg seroconversion rate was found to be 78.6% in 70 patients with a 6-month follow-up, which is consistent with the literature. In this study, the most commonly used antiviral agent was TDF (45.7%). Additionally, entecavir (38.3%), lamivudine (12.1%), and TAF (3.73%) were used.

Our study revealed that age was an independent predictor of HBsAg seroconversion. The chronic progression rate of HBV following acute hepatitis B infection may increase in older patients due to immunosenescence [28]. It is the decline in immune system function associated with aging [29]. It affects both innate and adaptive immunity, causing a decrease in T cells and the production of neutralizing antibodies [30]. However, studies investigating the relationship between age and HBsAg seroconversion rate are insufficient in AHB patients receiving antiviral therapy. In contrast to our study, a multicenter cohort study of 212 AHB patients from Japan found that age was not relevant for progression to chronicity [15]. This result may be related to the difference in the age distribution of the patients. In addition, the HBsAg seroconversion rate was found to be lower in the patients with DM in our study. Diabetes mellitus may increase the risk of HBV chronicity by reducing T cell function and impairing liver metabolism [31].

The severity of liver damage in acute hepatitis B is related to the strength of the host immune response [32]. The clinical and laboratory findings of patients progressively deteriorate due to immune-mediated lysis of infected hepatocytes [32,33]. However, in these patients, excessive immune response facilitates HBsAg seroconversion and contributes to the termination of infection [33]. In our study, loss of appetite, AST, PT, and INR elevation were found to be higher in the patients with HBsAg seroconversion, which is thought to be related to excessive immune response. In a recent study, ALT levels were found to be higher in the patients with HBsAg seroclearance within six months, suggesting a similar mechanism [15].

In the literature, studies investigating the effect of the choice of antiviral drugs on the HBsAg seroconversion rate are controversial. In a recent study comparing lamivudine versus entecavir, Cercel et al. revealed that the patients treated with entecavir demonstrated a better response in terms of HBsAg seroconversion [25]. In contrast, in another study which used entecavir, TDF, and lamivudine for AHB treatment, Jochum et al. revealed that the choice of antiviral drugs in these patients did not affect the HBsAg seroconversion rate [14]. In our study, TDF, entecavir, lamivudine, and TAF were used, and no difference was found between the antiviral agents in terms of HBsAg seroconversion. However, in our study, antiviral agents were selected according to their availability, and the number of these agents was limited. In addition, the timing of antiviral treatment initiation did not influence HBsAg seroconversion in our study. Similarly, recent studies reported that the time from diagnosis to initiation of antiviral treatment did not affect the HBsAg seroconversion rate [14,21].

In this study, clinical deterioration was observed in five patients despite antiviral treatment. One patient underwent liver transplantation, and one patient died while waiting for a suitable donor. In a recent study conducted in our country, 2 of 24 patients with severe AHB infection who received antiviral treatment had clinical deterioration. Similarly, one patient received a liver transplant, and another patient died while waiting for a suitable donor [27]. Furthermore, the mortality rate in this study was similar to the results in previous studies of patients with AHB treated with antiviral therapy [14,21,25].

### Limitation

This is a retrospective study including a total of 107 patients. Unfortunately, a total of 37 patients were lost to follow-up before six months, and these patients were not included in the comparison due to HBsAg seroconversion. It may be useful to evaluate the comparison with a large number of patients. In our study, due to the limited number of non-seroconversion events, the events per variable (EPV ≥ 10) rule could not be fully met in the multivariate logistic regression analysis. This may have increased the risk of overfitting and unstable estimates. The results should therefore be interpreted cautiously, taking this limitation into account.

## 5. Conclusions

Age was an independent predictor of HBsAg seroconversion in AHB patients receiving antiviral treatment. This study emphasizes the importance of follow-up of elderly patients in terms of chronicity after discharge.

## Figures and Tables

**Table 1:** Baseline characteristics of the patients.

	All Patients (*n* = 107)
Age (years), median (IQR)	48 (37–58.5)
Gender (male), *n* (%)	71 (66.3)
Smoke, *n* (%)	36 (33.6)
Alcohol use, *n* (%)	10 (9.34)
Comorbidities, *n* (%)	
Hypertension	22 (20.5)
Diabetes mellitus	15 (14)
CAD	14 (13)
Malignancy	9 (8.41)
Neurologic disease	7 (6.54)
COPD	5 (4.67)
Symptoms at hospital admission *n* (%)	
Fatigue	92 (85.9)
Loss of appetite	73 (68.2)
Nausea, vomiting	61 (57)
Jaundice	20 (18.6)
Abdominal pain	11 (10.2)
Pruritus	3 (2.80)
The length of the symptoms prior to antiviral treatment, (day) median (IQR)	13 (8–20)
Total length of hospital stay, (day) median (IQR)	16 (9.5–27)
Laboratory values median (IQR)	
Leukocyte count (/mm^3^)	6565 (5450–8685)
Hemoglobin (g/dL)	13 (12–14.8)
Platelet count (/mm^3^)	199,000 (161,000–263,000)
ALT (U/L) (6–41)	1553 (964.2–2133)
AST (U/L) 6–40)	1188 (691.7–1722)
GGT (U/L) (0–60)	151 (97.2–289)
ALP (U/L) (40–129)	174.5 (141.5–226.7)
LDH (U/L) (135–225)	454 (350–672)
Total bilirubin (mg/dL) (0.2–1.2)	11.9 (7–16.3)
Direct bilirubin (mg/dL) (0–0.3)	7.98 (5.4–12.4)
Prothrombin time (s) (10–14.5)	16 (13.4–19.6)
INR (0.8–1.2)	1.4 (1.2–1.6)
Albumin (mg/dL) (34–44)	34 (30–38)
Creatinine (mg/dL) (0.7–1.2)	0.75 (0.6–0.9)
Sodium (mmol/L) (136–145)	135 (133–137)
HBV-DNA (IU/mL)	4,512,525 (620,150–20,429,536)
Indications for antiviral treatment, *n* (%)	
Prolonged INR	42 (39.2)
Persistent symptoms	20 (18.6)
Acute liver failure	16 (14.9)
Hyperbilirubinemia	15 (14)
The median duration of antiviral therapy, median (IQR)	32 (28–90)
Antiviral treatment, *n* (%)	
TDF	49 (45.7)
Entecavir	41 (38.3)
Lamivudine	13 (12.1)
TAF	4 (3.73)
Supportive care, *n* (%)	
Proton pump inhibitors	42 (32.7)
Lactulose	24 (22.4)
Vitamin K	15 (14)
BCAAs	13 (12.1)
Ursodeoxycholic acid	11 (10.2)
Fresh frozen plasma	4 (3.73)
Unfavorable outcomes, *n* (%)	
Progression of HE	5 (4.67)
Liver transplantation	1 (0.93)
Died	2 (1.86)

Note: IQR, interquartile range; CAD, coronary artery disease; COPD, chronic obstructive pulmonary disease; ALT, alanine aminotransferase; AST, aspartate aminotransferase; GGT, gamma-glutamyl transferase; ALP, alkaline phosphatase; LDH, lactate dehydrogenase; INR, international normalized ratio; HBV, hepatitis B virus; TDF, tenofovir disoproxil fumarate; TAF, tenofovir alafenamide fumarate; BCAAs, branched-chain enriched amino acid solution; HE, hepatic encephalopathy.

**Table 2:** Comparison of demographic and clinical characteristics according to HBsAg seroconversion.

	HBsAg Non-Seroconversion (*n* = 15)	HBsAg Seroconversion (*n* = 55)	*p*-Value
Age (years), median (IQR)	63 (57–72)	43 (36–57)	**<0.001**
Gender (male), *n* (%)	10 (66.7)	32 (58.2)	0.552
Smoking, *n* (%)	3 (20.0)	17 (30.9)	0.551
Alcohol use, *n* (%)	2 (13.3)	7 (12.7)	0.889
Comorbidities, *n* (%)			
Hypertension	10 (40.0)	10 (18.2)	0.074
Diabetes mellitus	5 (33.3)	6 (10.9)	**0.034**
CAD	-	9 (16.4)	0.098
Malignancy	3 (20.0)	3 (5.5)	0.108
Neurologic disease	-	4 (7.3)	0.372
COPD	1 (6.7)	3 (5.5)	0.628
Symptoms at hospital admission *n* (%)			
Fatigue	12 (80.0)	50 (90.9)	0.226
Loss of appetite	7 (46.7)	42 (76.4)	**0.026**
Nausea, vomiting	7 (46.7)	31 (56.4)	0.352
Jaundice	6 (40.0)	11 (20.0)	0.109
Abdominal pain	-	7 (12.7)	0.169
Pruritus	1 (6.7)	1 (1.8)	0.385
The length of the symptoms prior to antiviral treatment, (day) median (IQR)	12 (10–17)	14 (8–20)	0.959
Total length of hospital stay, (day) median (IQR)	20 (14–25)	16 (9–28)	0.450

Note: HBsAg, Hepatitis B surface antigen; IQR, interquartile range; CAD, coronary artery disease; COPD, chronic obstructive pulmonary disease.

**Table 3:** Laboratory results at the initiation of antiviral treatment.

Laboratory Values Median (IQR)	HBsAg Non-Seroconversion (*n* = 15)	HBsAg Seroconversion (*n* = 55)	*p*-Value
Leukocyte count (/mm^3^)	5590 (4900–9110)	6800 (5425–8685)	0.333
Hemoglobin (g/dL)	13.0 (12.0–14.1)	13.2 (12.0–14.8)	0.923
Platelet count (/mm^3^)	210,000 (182,000–226,000)	191,000 (154,000–270,000)	0.932
ALT (U/L) (6–41)	1464 (309–2025)	1577 (968–2001)	0.363
AST (U/L) 6–40)	712 (395–1316)	1265 (644–2022)	**0.048**
GGT (U/L) (0–60)	284 (151–440)	152 (99–293)	0.080
ALP (U/L) (40–129)	154 (135–210)	190 (152–233)	0.227
LDH (U/L) (135–225)	442 (358–501)	459 (343–718)	0.371
Total bilirubin (mg/dL) (0.2–1.2)	6.91 (4.47–15.74)	11.00 (6.95–16.40)	0.226
Direct bilirubin (mg/dL) (0–0.3)	5.4 (1.5–11.7)	7.8 (5.4–13.4)	0.114
Prothrombin time (s) (10–14.5)	14.3 (12.1–15.9)	16 (13.6–19.8)	**0.042**
INR (0.8–1.2)	1.14 (1.06–1.33)	1.4 (1.21–1.6)	**0.011**
Albumin (mg/dL) (34–44)	35 (30–38)	34 (32–39)	0.752
Creatinine (mg/dL) (0.7–1.2)	0.79 (0.60–0.86)	0.71 (0.60–0.90)	0.895
Sodium (mmol/L) (136–145)	136 (132–138)	136 (134–138)	0.771
HBV-DNA (IU/mL)	11,421,901 (2,167,614–63,127,998)	6,937,632 (732,698–57,900,000)	0.749

Note: HBsAg, Hepatitis B surface antigen; IQR, interquartile range; ALT, alanine aminotransferase; AST, aspartate aminotransferase; GGT, gamma-glutamyl transferase; ALP, alkaline phosphatase; LDH, lactate dehydrogenase; INR, international normalized ratio; HBV, hepatitis B virus.

**Table 4:** Treatment indications, antiviral agents, supportive care, and outcomes.

	HBsAg Non-Seroconversion (*n* = 15)	HBsAg Seroconversion (*n* = 55)	*p*-Value
Indications for antiviral treatment, *n* (%)			
Prolonged INR	6 (40.0)	21 (38.2)	0.562
Persistent symptoms	3 (20.0)	11 (20.0)	0.626
Acute liver failure	2 (13.3)	14 (25.5)	0.268
Hyperbilirubinemia	2 (13.3)	7 (12.7)	0.621
The median duration of antiviral therapy, median (IQR)	30 (22.5–60)	40.3 (30–120)	0.564
Antiviral treatment, *n* (%)			
TDF	6 (40.0)	29 (52.7)	0.382
Entecavir	5 (33.3)	21 (38.2)	0.730
Lamivudine	3 (20.0)	4 (7.3)	0.163
TAF	1 (6.7)	1 (1.8)	0.385
Supportive care, *n* (%)			
Proton pump inhibitors	3 (20.0)	26 (47.3)	0.057
Lactulose	5 (33.3)	13 (23.6)	0.446
Vitamin K	3 (20.0)	6 (10.6)	0.294
BCAAs	2 (13.3)	5 (9.1)	0.468
Ursodeoxycholic acid	-	6 (10.9)	0.221
Fresh frozen plasma	-	2 (3.6)	0.615
Unfavorable outcomes, *n* (%)			
Progression of HE	-	3 (5.9)	0.455
Liver transplantation	-	1 (1.8)	0.756
Died	-	1 (1.8)	0.756

Note: HBsAg, Hepatitis B surface antigen; INR, international normalized ratio; TDF, tenofovir disoproxil fumarate; TAF, tenofovir alafenamide fumarate; BCAAs, branched-chain enriched amino acid solution; HE, hepatic encephalopathy.

**Table 5:** Multivariate logistic regression analysis of variables associated with HBsAg seroconversion status.

	B	Wald	Exp(B)	95% CI for EXP (B)		*p*-Value
				Lower	Upper	
Age (years)	−0.077	6.863	0.926	0.874	0.981	**0.009**
Diabetes mellitus	−1.741	3.395	0.175	0.028	1.117	0.065
Loss of appetite	1.397	3.033	4.041	0.839	19.459	0.082
AST (U/L)	0.001	2.566	1.001	1	1.003	0.109
Prothrombin time (s)	−0.009	0.012	0.991	0.847	1.16	0.914

Note: HBsAg, Hepatitis B surface antigen; CI, confidence interval; AST, aspartate aminotransferase.

## Data Availability

The raw data supporting the conclusions of this article will be made available by the authors on request.
